# Telegerontology as a Novel Approach to Address Health and Safety by Supporting Community-Based Rural Dementia Care Triads: Randomized Controlled Trial Protocol

**DOI:** 10.2196/resprot.8744

**Published:** 2018-02-22

**Authors:** Elizabeth M Wallack, Chelsea Harris, Michelle Ploughman, Roger Butler

**Affiliations:** ^1^ Recovery and Performance Lab Faculty of Medicine Memorial University of Newfoundland St John's, NL Canada; ^2^ Discipline of Family Medicine Faculty of Medicine Memorial University of Newfoundland St John's, NL Canada

**Keywords:** Aging, Remote Assessment, Monitoring

## Abstract

**Background:**

Telegerontology is an approach using videoconferencing to connect an interdisciplinary team in a regional specialty center to patients in rural communities, which is becoming increasingly practical for addressing current limitations in rural community-based dementia care.

**Objective:**

Using the remotely-delivered expertise of the Telegerontology dementia care team, we aim to enhance the caregiver/patient/physician triad and thereby provide the necessary support for the person with dementia to “age in place.”

**Methods:**

This is a cluster randomized feasibility trial with four rural regions in the province of Newfoundland and Labrador, Canada (2 regions randomly assigned to “intervention” and 2 to “control”). The study population includes 22 “dementia triads” that consist of a community-dwelling older Canadian with moderate to late dementia, their family caregivers, and their Primary Care Physician (PCP). Over the 6-month active study period, all participants will be provided an iPad. The intervention is intended as an adjunct to existing PCP care, consisting of weekly Skype-based videoconferencing calls with the Telegerontology physician, and other team members as needed (occupational therapist, physical therapist etc). Control participants receive usual community-based dementia care with their PCP. A baseline (pre-) assessment will be performed during a home visit with the study team. Post intervention, 6- and 12-month follow-up assessments will be collected remotely using specialized dementia monitoring applications and Skype calls. Primary outcomes include admission to long-term care, falls, emergency room visits, hospital stays, and caregiver burden.

**Results:**

Results will be available in March of 2018.

**Conclusions:**

Results from this study will demonstrate a novel approach to dementia care that has the potential to impact both rural PCPs, family caregivers, and people with dementia, as well as provide evidence for the utility of Telegerontology in models of eHealth-based care.

## Introduction

In 2011 the estimated prevalence of dementia in Canada was 340,000 with just under one-third (109,500) living at home [1]. Community-based dementia care relies heavily on family caregivers, with 92% of surveyed Canadians with dementia reporting friends or family members managing care and transportation. A further 58%-92% report family caregivers assisting with personal care, medical care, activities of daily living, meal preparation, and emotional support [1]. With such high demands on family caregivers of people with dementia, they are at increased risk of stress and burden [2], medical and psychiatric comorbidities [3,4], and social isolation [5]. Rates of dementia [6] and mortality [7] are also higher among caregivers, perpetuating and compounding the issue into a cycle of caregiving that leads to early disability, cognitive decline, and death. These detrimental effects of caregiving can also translate to negative consequences for people with dementia, including earlier institutionalization [8] and increased mortality [9]. Approaches that support the caregiver as well as the patient are therefore fundamental to community-based dementia care.

The health care triad model of dementia care proposes management of dementia that is both family and patient centered, while acknowledging that the primary care physicians (PCPs) are usually the first contact in the health care system for persons with dementia and their family caregivers [10]. This integrative approach values symptom diagnosis and management, medication management, and emotional support with a focus on comprehensive care coordination [10] and has been employed in both rural and urban settings [11]. And while this is a favorable approach, PCPs in rural Canada can face challenges when attempting to provide adequate community-based care for dementia patients and their families. Although rural Canadian PCPs are more accessible than specialist services, some research suggest that they may have limited training in dementia specific management [12]. Rural PCPs also report a need for better collaboration, partnerships, and integration of services [13] in order to provide the very best care for people with dementia and their families. How best to support the dementia triad in rural areas of Canada is still poorly understood, and few studies have explored the utility of the health care triad model of dementia care as a theoretical basis for an intervention [11]. Taken together, this evidence demonstrates a clear need to design and deliver evidence-based services to support people living with dementia in the community, their family caregivers, and PCPs [14].

Technology-based intervention is one line of inquiry aiming to address support for rural dementia care triads. There is some evidence to suggest that people with dementia and their family caregivers are already using the Internet to access Internet-based health resources [15]. Internet interventions for family caregivers of people with dementia have shown promise addressing caregiver well-being [16-18]. Less is known about how the Internet can be used to manage dementia at home. Some research suggests that videoconferencing is a valid method to assess dementia [19,20]. Telemedicine has been used to accurately diagnose dementia [21] and telehealth has effectively supported pre-assessment and follow-up when used in combination with an in-person interdisciplinary memory clinic assessment [22].

This paper describes a novel approach to rural community-based dementia care that combines the health care triad model of dementia care (patient, caregiver, and PCP) with an innovative Telegerontology intervention. Telegerontology is an approach using videoconferencing to connect an interdisciplinary team in a regional specialty center to people in rural communities. The aim of this study is to use Telegerontology in order to systematically detect and address care needs of people with dementia and their family caregivers, while augmenting PCP relationships with dementia patients and their families. The overall objective is to assist people with dementia to age successfully “in place.” Furthermore, findings from this study will be used to identify feasibility issues that will inform the design of a full-scale randomized control trial (RCT).

## Methods

### Aim

Using the remotely delivered expertise of the Telegerontology dementia care team, we aim to enhance the caregiver/patient/physician triad and thereby improve rural community-based care for people with dementia.

### Objectives

The objectives will be as follows:

1. Test Telegerontology’s ability to enhance the caregiver/patient/ PCP triad and thereby improve care “in place” (ie, health care utilization) for people with dementia.
2. Assess and optimize a remote monitoring method for managing care of people with moderate- to late-stage dementia living at home in rural Canada in order to inform the design of a full-scale RCT.
3. Explore issues related to care of people with dementia from the caregivers’ and PCPs’ perspectives.

### Study Design

#### Overview

The study was approved by the local research ethics authority. This is a cluster randomized feasibility trial assessing 4 rural regions (2 per study arm) comparing usual rural community-based dementia care to Telegerontology plus usual rural community-based dementia care. Using the opaque envelope method, the rural regions will be randomized to control or intervention after baseline assessment. The intervention will last 6 months with the measurement of outcomes taking place at baseline, after 6 months (post), with follow-up occurring 6 months (6-month follow-up) and 12 months (12-month follow-up) after cessation of the intervention.

The study population will include 22 “dementia triads” that consist of a community-dwelling older Canadian with moderate to late dementia, their family caregivers, and their PCP. The study will be carried out in communities located in rural areas of Newfoundland and Labrador, Canada but will be monitored remotely from the metropolitan city of St John’s, Newfoundland and Labrador. We define *rural* in this study to mean a community with less than 10,000 residents and a location that is greater than two hours’ drive from the metropolitan city, where specialist health services are located. Before randomization, recruitment will begin by enrolling PCPs from rural communities who will make initial contact with potential participants. Written informed consent will be obtained for every individual agreeing to participate in the study except in the case for an individual with advanced dementia who lacks capacity, in which case consent will be obtained from the substitute decision maker. Consent will be reviewed without the presence of the PCP in order to limit undue influence for participation in the study.

#### Inclusion Criteria

##### Dementia Patient/Caregiver Dyad

Community-dwelling adults aged ≥60 years will be screened by PCP for having a score of 4 or greater on the Global Deterioration Scale [23], and have a formal diagnosis of dementia based on the Diagnostic and Statistical Manual of Mental Disorders IV. Caregiver is defined as an individual who self-identifies as providing support with instrumental activities of daily living in an informal (friend or family) capacity and who was referred to the study by the PCP.

##### PCPs

PCPs located in a rural area of Newfoundland and Labrador must be willing to participate in a 1 hour focus group, and must refer at least 1 community-dwelling patient with moderate/late-stage dementia who agrees to participate in the study.

#### Exclusion Criteria

##### Dementia Patient/Caregiver Dyad

Patients with a diagnosed comorbid psychiatric condition (eg, schizophrenia, bipolar disorder, psychoses not yet determined) will be excluded. In addition, those who are currently participating in other studies or experimental therapies will be excluded.

##### Dropout

Dropout is defined as any participant who voluntarily withdraws or who cannot be successfully contacted within a month of the defined follow-up times. The impact of dropouts on statistical analysis, including intention to treat, will be discussed subsequently.

#### Control and Intervention Group

Over the 6-month active study period, all participants will continue usual community-based dementia care with their PCPs. All participants will also be given new iPads by the study team with Skype and the specialized dementia monitoring applications installed. In addition, all participants will be referred to the Canadian Alzheimer Society’s FirstLink Program [24,25] which includes a 10-week education session for caregivers that can be accessed remotely by Skype, and provided links to Internet-based health resources for dementia caregivers. Because FirstLink is already available in the target communities, all participants were referred in order to standardize exposure across groups.

**Figure 1:**
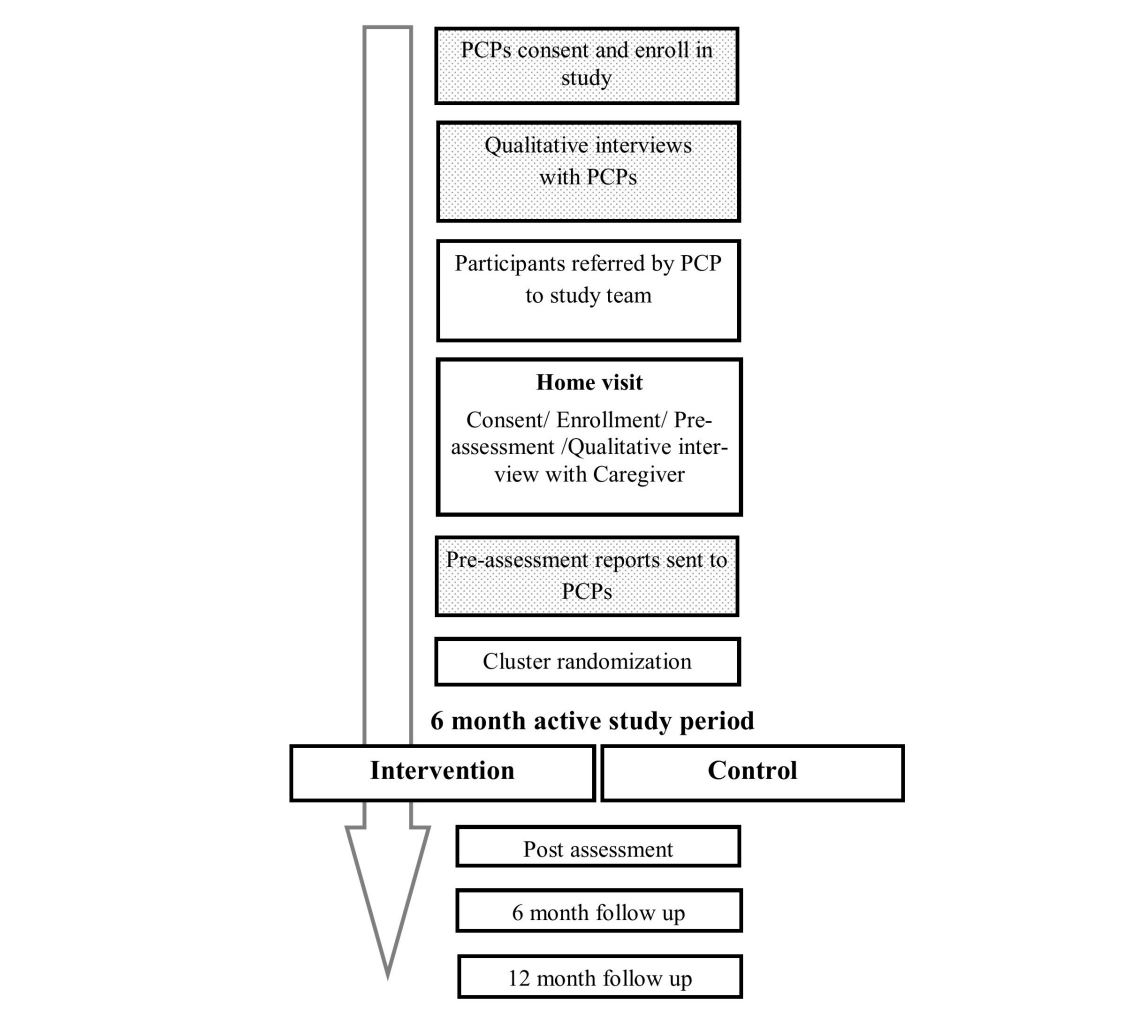
Study flow. After PCP recruitment, participant referrals and home assessment, the rural regions will be randomized to control or intervention.

PCPs of all participants will also receive a written report of the initial home assessment at the beginning of the 6-month active study period to implement at their discretion. Study flow is described in detail in Figure 1.

#### Intervention Group

The intervention works as an adjunct to existing PCP care and consists of scheduled weekly Skype-based videoconferencing calls (additional calls available as needed) with the Telegerontology physician, who is a family practice geriatrician. The program’s development was based on principals of the health care triad model of dementia care [10]. Calls will focus on symptom diagnosis and management, medication management, comprehensive care coordination, and emotional support. During the 6-month intervention, the Telegerontology physician will liaise via telephone and email correspondence with the PCP and specialized geriatric team as required. The team will include a geriatrician, geriatric psychiatrist, nurse, physiotherapist and occupational therapist. Access to other allied health professionals (social worker, speech language pathologist, and recreational therapist) will also be available when deemed necessary. During this time, participant families will also have access to specialized dementia monitoring iPad applications that may be used by the Telegerontology physician in clinical decision making. Any potential changes to patient’s treatment plans will be managed by the participant’s PCP in consultation with the Telegerontology physician and acting in collaboration with the specialist gerontology health care team.

### iPad Application Development

Three standardized scales to assess dementia and caregiver burden were developed into specialized monitoring apps for the iPad through Memorial University’s Distance Education, Learning and Teaching Support. The Cohen Mansfield Agitation Inventory [26], the Cornell Scale for Depression in Dementia [27] and the Caregiving Hassles Scale [28] were selected because they were designed to be completed by caregivers, and were appropriate to evaluate caregiver burden or domains of function for targeted management of moderate to late stage dementia. The scales are validated tools to assess agitation, depression and daily hassles associated with dementia care [28-30] and will be discussed in detail subsequently.

**Figure 2:**
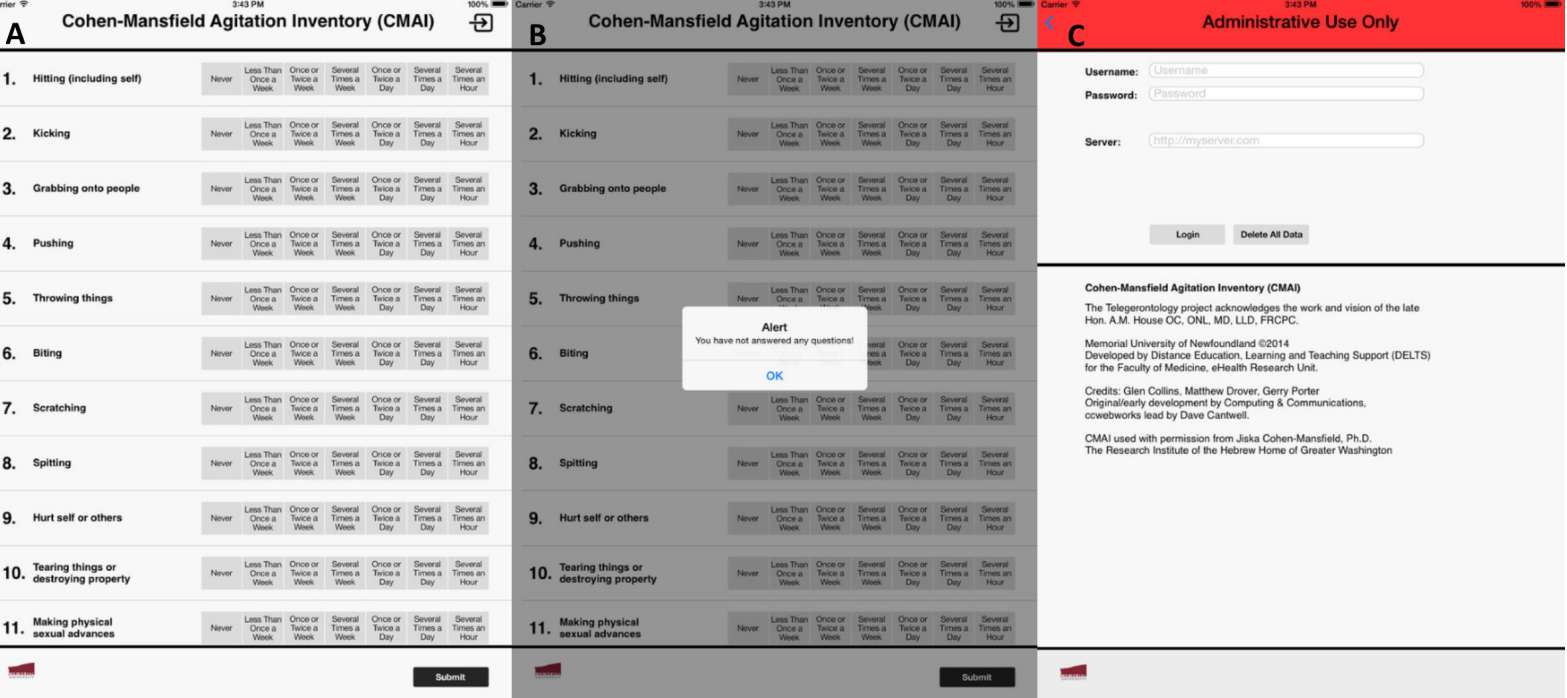
(A) Main screen display, (B) Error message for missing responses, (C) Administrative login page.

**Figure 3:**
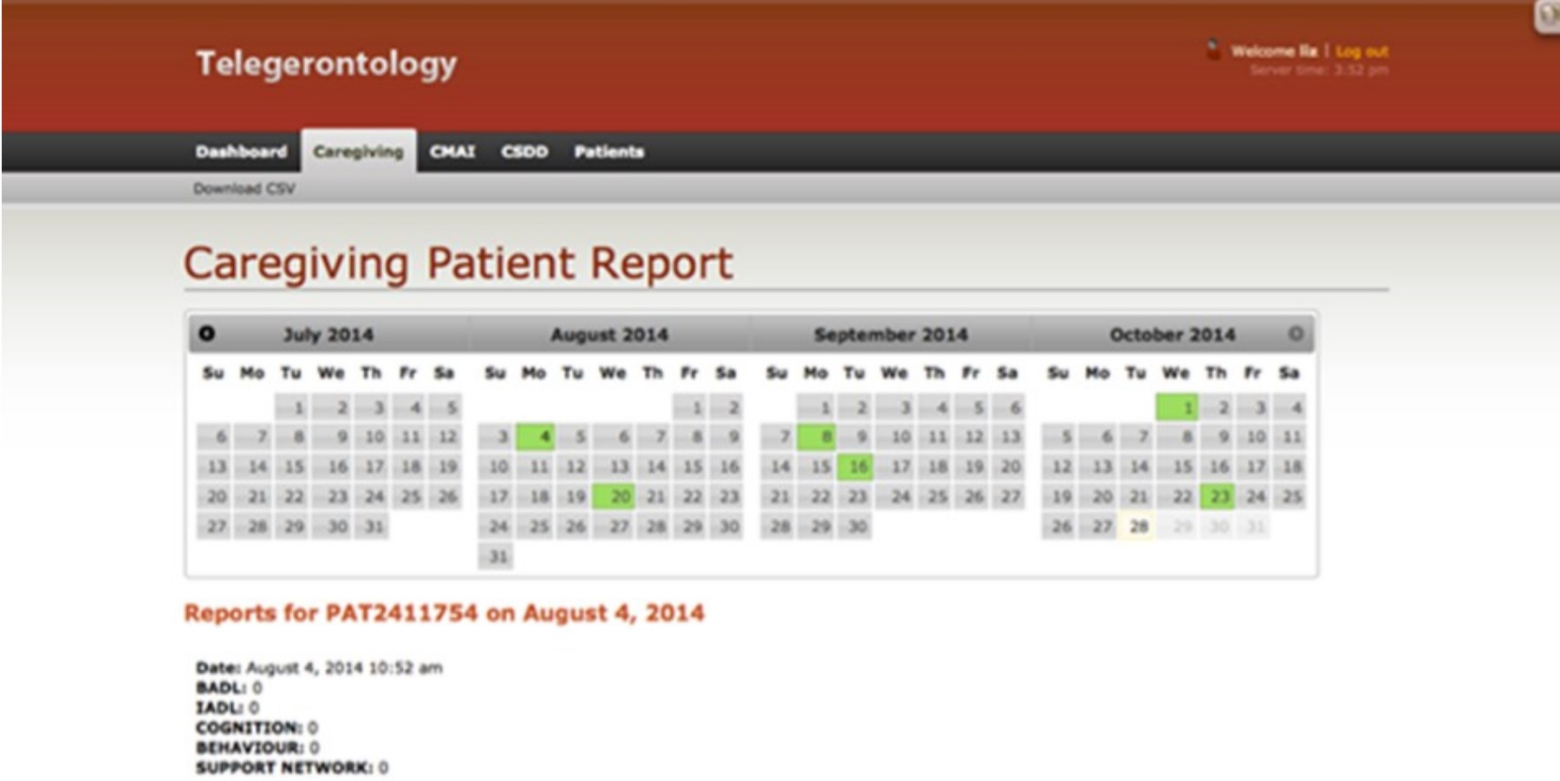
Screenshot of secure web portal. Colored calendar blocks indicate days when app is completed by caregiver. Scores from reports are displayed by clicking on a colored block.

The apps will be pre-loaded onto iPads. The main screen of the app displays a list of questions (Figure 2, panel A). An error message appears if participants miss questions and a list of missed questions appears to direct participants (Figure 2, panel B). Login is managed by the Telegerontology team, so participants will not be required to enter username, password or server information when opening the apps (Figure 2, panel C). Permission to reproduce these scales was granted by the authors and acknowledgements were included on the administrative page of the app (Figure 2, panel C). Each participant will be assigned a unique anonymous ID linked to the study number, which will allow the Telegerontology physician to access information remotely from a secure web portal (Figure 3).

### Telegerontology Measurement Framework

A measurement framework was designed to include domains from The International Classification of Functioning, Disability and Health [31] and the Wilson-Cleary Conceptual Model of Patient Outcomes [32]. Assessments encompass the following domains: personal, environment, impairment, disability, participation, quality of life, and health care use. Outcome measures, clinical assessment tools, remote monitoring tools and circle of care measures to be administered at each time point in relation to the study objectives are described in Table 1.

### Outcome Measures

Primary and secondary outcomes will be collected at baseline (pre-), during the intervention, after the intervention (post), and 6- and 12-month follow up. Assessments will be completed by trained clinical personnel who are also members of the research team. First assessments will be completed during the home visit, with subsequent measures collected remotely. All data will be manually entered into SPSS for analysis. The study outcomes will be used to answer the primary research question regarding Telegerontology’s ability to enhance the caregiver/patient/PCP triad and thereby improve care “in place” for people with dementia.

#### Primary Outcomes

Primary outcomes will be reported by caregivers and confirmed through medical records and chart reviews (schedule of assessment described in Table 1). Based on Canadian incidence rates, we anticipate these outcomes will be detectable during the 18-month study period [33-35]. The primary outcomes will be as follows:

1. Admission to long-term care (LTC): Date that participant is admitted to LTC.
2. Emergency room (ER) visits: Total number of ER visits over 6 months.
3. Falls: Total number of falls over 6 months.
4. Hospital stays: Total number of hospital stays over 6 months.
5. Caregiving Hassles Scale: This 42-item scale assesses the daily burden of caring for a family member with dementia. Caregivers are asked to rate patient requirements and behaviors on a 5-point scale of frequency, ranging from “Did not occur” to “A lot.” High scores indicate higher level of caregiver burden. Assessment takes 20 minutes to complete [28]. Administered by iPad, at pre, post, 6- and 12-month measures, in addition to an outcome measure, this scale will be used as a monitoring tool during the intervention at the discretion of the Telegeronology physician.

#### Secondary Outcomes

The secondary outcomes will be as follows:

1. Referrals: Total number of referrals to specialist care over 6 months.
2. PCP visits: Total number of PCP visits over 6 months.

### Clinical Assessment Tools

These tools will be used to help characterize the progression of dementia throughout the study and to inform clinical decision making (schedule of assessment described in Table 1). These assessments will also be used to address feasibility issues and to inform remote monitoring practices. All assessments will be administered by the physician or physiotherapist.

#### Demographics and Medical History

Demographic information including age, sex, marital status, educational background, and living situation will be collected along with information about the patient’s circle of care. Medical information including allergies, comorbid diagnoses, medications, consultations, laboratory reports, details of the dementia diagnosis, and results of previous tests and assessments will be collected by chart review.

**Table 1:** Measures mapped to study objectives and schedule of assessment.

Measure		Schedule (Stage)									
		Baseline		During intervention		Post assessment		6-month follow-up		12-month follow-up	
**Outcome Measures**											
	Emergency room visits^a^						✓^b^		✓		✓
	Hospital stays^a^						✓		✓		✓
	Admission to LTC^a,c^						✓		✓		✓
	Falls^a^						✓		✓		✓
	Referrals^a^						✓		✓		✓
	PCP visits^a,d^						✓		✓		✓
	Caregiving Hassles Scale^a,e^		✓		✓		✓		✓		✓
**Clinical Assessment Tools**											
	Demographics and medical history^e^		✓								
	Barthel Index of Activities of Daily Living^e^		✓								
	Timed Up and Go test^e^		✓								
	Kettle test^e^		✓								
	Face washing functional task^e^		✓								
	Custom Caregiver Quality of Life Index^e^		✓								
	Global Deterioration Scale^e^		✓				✓		✓		✓
	Mini Mental Status Exam^e^		✓				✓		✓		✓
	Montreal Cognitive Assessment^e^		✓				✓		✓		✓
	Occupational therapy home video assessment^e^		✓								
	Cohen Mansfield Agitation Inventory^e^		✓		✓		✓		✓		✓
	Cornell Scale for Depression in Dementia^e^		✓		✓		✓		✓		✓
	Telegerontology time tracking^e^				✓						
	Qualitative interviews (caregivers and PCPs)^f^		✓								

^a^Test Telegerontology’s ability to enhance the caregiver/patient/ PCP triad and thereby improve care ‘in place’ (ie, health care utilization) for people with dementia.

^b^✓=Performed at time point.

^c^LTC: Long-term care.

^d^PCP: Primary care physician.

^e^Assess and optimize a remote monitoring method for managing care of people with moderate- to late- stage dementia living at home in rural Canada in order to inform the design of a full-scale randomized control trial.

^f^Explore issues related to care of people with dementia from the caregivers’ and primary care physicians’ perspectives.

#### Barthel Index of Activities of Daily Living

The Barthel Index assesses self-care, mobility, and activities of daily living. It is widely used in geriatric assessment settings. Information is collected from observation, self-report or informant report. It takes approximately 5-10 minutes to complete. Maximum score is 100, indicating complete independence. Low scores on individual items highlight areas of need [36].

#### Timed Up and Go Test

The Timed Up and Go test assesses mobility, balance, walking ability, and fall risk in older adults [37]. When the assessment begins, the patient sits in the chair with his/her back against the chair back. On the command “go”, the patient rises from the chair, walks 3 meters at a comfortable and safe pace, turns, walks back to the chair, and sits down. Timing begins at the instruction “go” and stops when the patient is seated. The time (sec) between the command to start and when the buttocks touch the chair is collected. The patient should have one practice trial that is not included in the score and must use the same assistive device each time he/she is tested to be able to compare scores. The Timed Up and Go takes less than 5 minutes to administer [37]. Minimal detectable change in Alzheimer’s disease has been reported as 4.09 seconds [38].

#### Kettle Test

The Kettle Test was developed as a brief performance-based measure to assess cognitive skills in a functional context. The test uses a standard set of items including an electric kettle (empty and disassembled parts); ingredients for beverages, presented on a tray together with other ingredients as distracters; and necessary dishes and utensils together with distracters. The task of preparing two hot beverages is broken down into 13 discrete steps that can be evaluated. All 13 steps of the task are scored on a 4-point scale. The total score ranges from 0 to 52 with higher scores indicating the need for greater assistance [39].

#### Face Washing Functional Task

The face washing task is not based on a standard scale. Participants will be asked to demonstrate for the observer the personal care activity of face washing. Qualitative observations will be noted, focusing on aspects of safety and independence.

#### Global Deterioration Scale

This scale assesses the stages of cognitive function in primary degenerative dementia. It is broken down into 7 different stages. Stages 1-3 are the mild stages of dementia. Stages 4-5 are the moderate stages of dementia. Beginning in stage 6, an individual can no longer maintain independence without assistance. Within the Global Deterioration Scale , each stage is numbered (1-7), given a short title (Mild, Moderate, etc), and followed by a brief listing of the characteristics for that stage. Caregivers can estimate the stage of an individual in the disease process by observing that individual's behavioral characteristics and comparing them to the Global Deterioration Scale [23].

#### Mini Mental Status Exam

The Mini Mental Status Exam (MMSE) is an 11-question measure that tests five areas of cognitive function: orientation, registration, attention and calculation, recall, and language. The maximum score is 30. A score of less than 24 is indicative of cognitive impairment. The MMSE takes about 5-10 minutes to administer and is therefore practical to use repeatedly and routinely. The instrument relies heavily on verbal response and reading and writing. Therefore, patients that are hearing and visually impaired, intubated, have low English literacy, or those with other communication disorders may perform poorly even when cognitively intact [40].

#### Montreal Cognitive Assessment

The Montreal Cognitive Assessment (MoCA) test is a one-page 30-point test administered in approximately 10 minutes [41]. The MoCA assesses several cognitive domains including short-term memory, visuospatial abilities, executive functions verbal fluency and abstraction, attention, concentration and working memory, and orientation to time and place. The MoCA is more sensitive to mild cognitive impairment compared to the MMSE, and has also been used to assess cognitive impairment in Alzheimer’s disease [42], frontotemporal [43], and vascular dementia [44].

#### Custom Caregiver Quality of Life Index

The custom caregiver quality of life index includes 10 questions about the impact of caring for a person with dementia, aspects of the caregiver’s life, and the caregiver’s mental well-being. Areas include alteration in daily routine, sleep, financial train, outlook, mental stain, guilt, frustration, impact of illness on family, and feeling well informed about dementia. Scores range from 1 (not at all) to 5 (very much) with higher scores indicating greater impact of caregiving on quality of life.

### Remote Assessment Tools

The remote assessment tools will be used by the Telegerontology team for clinical decision making through the intervention period (schedule of assessment described in Table 1). We anticipate some variation in the frequency of their administration, dependent on the individual.

#### Cohen Mansfield Agitation Inventory

The Cohen Mansfield Agitation Inventory is a caregiver’s rating questionnaire consisting of a list of 29 agitated behaviors, each rated on a 7-point scale of frequency. Ratings pertain to the two weeks preceding the administration of the Cohen Mansfield Agitation Inventory [26] and takes approximately 20 minutes to complete.

#### Cornell Scale for Depression in Dementia

The Cornell Scale for Depression and Dementia was developed to assess signs and symptoms of major depression in patients with dementia and measure caregivers’ rating of 19 items. Ratings are based on symptoms present in the past week. Each question is scored as: 0=absent; 1=mild or intermittent; 2=severe; and n/a=unable to evaluate. High scores indicate more symptoms of depression [27].

#### Caregiving Hassles Scale

The Caregiving Hassles Scale has been described above under primary outcomes.

#### Occupational Therapy Home Video Assessment

The research assistant will collect a detailed video of the home and living space based on a standard checklist developed by the specialized gerontology health care team’s Occupational Therapist (Table 2). The Occupational Therapist will then assess the video recordings and compile a detailed report of home safety recommendations for the PCP to review with the patient/caregiver (schedule of assessment described in Table 1).

### Time Tracking

#### Telegerontology Time Tracking

Telegerontology team members will log the time spent delivering support to families during the intervention period as well as all other related tasks such as chart review and report writing. This measure will be used to help address feasibility issues, to inform remote monitoring practices, and may also inform future cost-benefit analyses based on the study findings.

### Circle of Care Measures

Circle of care is defined as anyone who is involved in patient care and can include formal care providers and informal caregivers [45,46]. These measures will not be used as outcome measures, but rather to address the research objective to identify existing issues in rural areas when caring for people with dementia from the caregivers’ and primary care physicians’ perspectives (schedule of assessment described in Table 1).

#### Baseline Interviews

Baseline interviews will be completed in person by the research assistant who will visit participants, caregivers and PCPs in their respective communities. All interviews will be audio recorded and transcribed for analysis. The interviewer will be experienced with qualitative research methods and will attempt to elicit detail using reflexive probes based on the participants’ answers.

#### PCP Interview

Prior to the active study phase, all recruited PCPs will be asked to take part in a qualitative focus group. The interview will center around 3 main questions (Textbox 1).

#### Caregivers Interview

All caregivers will be asked to take part in a qualitative interview at baseline. The interview will center around two main questions (Textbox 1).

### Sample Size Calculation

Sample size was calculated based on regional population statistics rather than for statistical power, targeting 2.5% of the population of community-dwelling dementia patients in the target region. Based on prevalence estimates, 98.5/1000 Canadians over 40 years of age have dementia with 34% living at home. The total population for the entire recruitment region of the present study is approximately 38,490 suggesting that the total population of community-dwelling dementia patents is approximately 1,289. Therefore, we aim to recruit 42 participants for inclusion in this study accounting for a 30% dropout rate.

**Table 2:** Occupational Therapy Home Safety Video checklist.

Video	Description
1	A short video walking from the driveway outside, going into the house through the usual entry door, straight through the kitchen/living area into the person's bedroom and then into the bathroom.
2	Kitchen (short video)
3	Bedroom (short video)
4	Bathroom (short video)
5	Living room (short video)
6	Stairs (short video)
7	Laundry (short video)

**Qualitative interview questions.**

PCP Interview Questions

- Describe the care required to manage people with dementia in their homes.
- From your point of view, how well are your patients being managed at home?
- How would you envision an ideal circle of care to maintain people with dementia in their homes?

Caregivers Interview Questions

- From your point of view, how well are you managing the health of your loved one at home?
- In an ideal world, how could things be improved? What are the main challenges you experience? Have you had successes caring for your loved one?

### Statistical Analysis

Data entry, consistency check, and cleaning will be performed prior to analysis. Mean and standard deviation will be used for continuous variables, while frequency and percentages will be used to describe categorical variables. We do not anticipate this study will detect statistically significant findings however, as part of a secondary analysis, unpaired *t* tests will be used. Differences between intervention and control groups with respect to outcomes will be compared using repeated measures ANOVA or independent *t* test split by group. Data analysis will be conducted using SPSS statistical software. A *P*<0.05 will be used to determine potential significance. The 95% CI for differences will also be calculated. With respect to dropout, every person who is randomized regardless or subsequent fall, death, or admission to LTC will be included in the final analysis. If participants drop out before the intervention or voluntarily withdraw they will not be included in the final analysis. For analysis of the clinical assessments tracked over the course of the study (schedule of assessment described in Table 1), intention-to-treat analysis will be used by carrying the last observation forward.

Qualitative analysis will follow the Framework Method [47]. This method helps build rigor and trustworthiness in qualitative analysis by providing a clear audit trail from the original data to the final themes including illustrative quotations.

## Results

Recruitment began in January of 2014. Data collection was completed in April 2017. Results will be available in March of 2018.

## Discussion

### Significance of Study

The proposed study is the first study, to our knowledge, to use Telegerontology together with Skype and specialized remote monitoring apps for the iPad to optimize health and safety among rural community-dwelling Canadians with dementia. The results could have particular importance given the international increase in prevalence of dementia [48], and the shift away from institutional-based models of care in Canada and other countries [49].

### Study Strengths and Limitations

The proposed study has several notable methodological strengths. The initial home visit is designed to help build rapport between the Telegerontology physician and participants. This method is also in keeping with previous research, which suggests that telehealth has effectively supported assessment and follow-up when used in combination with an in-person interdisciplinary memory clinic assessment [12].

Next, the Telegerontology measurement framework, based on the ICF and Wilson-Cleary models provides in-depth, multi-factor profiles of participants contributing to a methodology that acknowledges the broad range of factors that can impact health planning and management of community dwelling people with dementia. Likewise, the inclusion of caregivers and PCPs as participants adds further depth to both characterizing and assessing participants with dementia by acknowledging and building on the role of the caregiver and PCP in the health care triad. The development of remote monitoring apps from existing validated scales to assess dementia and caregiver burden is also a methodological strength of this study.

There are also several limitations in the protocol. The relatively small sample size can be seen as a drawback, however, based on regional prevalence estimates, the sample size is realistic for the target population. There may also be considerable variability between participants within a group, further complicating statistical analysis. Next, the Telegerontology physician serves as both an interventionist and assessor. This has the potential to create bias, however the primary outcome measures were selected partially to address this issue since they are based on participant self-report/chart review rather than clinical assessment.

### Conclusion

This proposed study will provide an evaluation of Telegerontology together with Skype and specialized remote monitoring apps for the iPad to optimize health and safety among rural community-dwelling Canadians with dementia. Results from this study will demonstrate a novel approach to dementia care that has the potential to impact both rural PCPs, family caregivers, and people with dementia, as well as provide evidence for the utility of Telegerontology in a full scale RCT.
